# Optimization in Visual Motion Estimation

**DOI:** 10.1146/annurev-vision-101623-025432

**Published:** 2024-09-19

**Authors:** Damon A. Clark, James E. Fitzgerald

**Affiliations:** 1 Department of Molecular, Cellular, and Developmental Biology, Yale University, New Haven, Connecticut, USA; 2 Department of Neurobiology, Northwestern University, Evanston, Illinois, USA; 3 Janelia Research Campus, Howard Hughes Medical Institute, Ashburn, Virginia, USA

**Keywords:** motion estimation, optimization, Bayes optimality, efficient coding, task optimization

## Abstract

Sighted animals use visual signals to discern directional motion in their environment. Motion is not directly detected by visual neurons, and it must instead be computed from light signals that vary over space and time. This makes visual motion estimation a near universal neural computation, and decades of research have revealed much about the algorithms and mechanisms that generate directional signals. The idea that sensory systems are optimized for performance in natural environments has deeply impacted this research. In this article, we review the many ways that optimization has been used to quantitatively model visual motion estimation and reveal its underlying principles. We emphasize that no single optimization theory has dominated the literature. Instead, researchers have adeptly incorporated different computational demands and biological constraints that are pertinent to the specific brain system and animal model under study. The successes and failures of the resulting optimization models have thereby provided insights into how computational demands and biological constraints together shape neural computation.

## 1. INTRODUCTION

Visual motion detection is a fundamental faculty in sighted animals, and it underlies many critical behaviors (Rust et al. 2006, Wei 2018, Yang & Clandinin 2018). To detect motion, the visual system must integrate information over time and space. This integration is requisitely nonlinear (see the sidebar titled Motion Detection Requires a Nonlinear Operation), which leads to surprising neural responses and offers rich theoretical opportunities. Motion across the retina is governed by straightforward geometry, making theoretical studies of the neural computation more tractable. All of these factors, together with years of study across visual systems in animals from flies to humans, have made visual motion detection a canonical neural computation.

**Figure 1:**
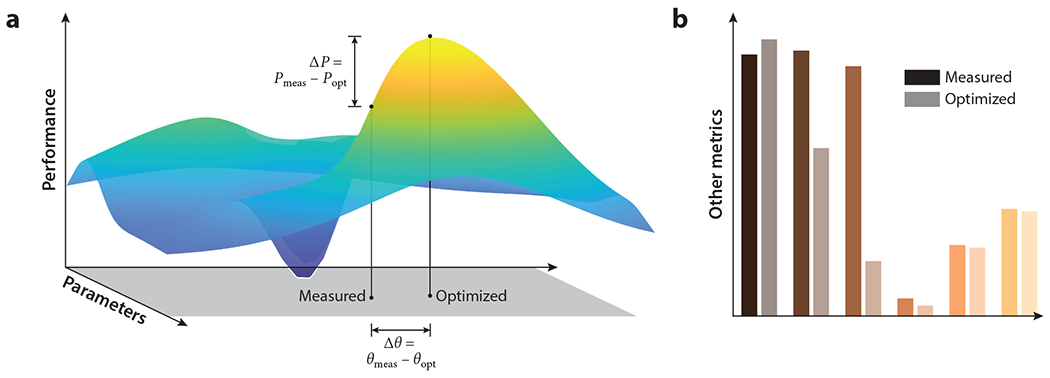
An optimized model acts as a concrete point of comparison for understanding the performance and features of the system. (*a*) The performance of an optimized model may be compared to the measured system’s performance (Δ*P*). The parameters of the optimized model may also be compared to those fit to the measured system (Δ*θ*). (*b*) One may also compare the measured system to the optimized model in terms of functional properties beyond the optimized one.

Over the course of evolution, natural selection has acted on sensory circuits, and one broad theory posits that these circuits may be viewed as optimized under some constraints (Barlow 1961, Sterling & Laughlin 2015). This sort of theory is useful because it presents a framework for understanding the function of neural circuits in terms of their performance (Figure 1). One may ask how a circuit performs a certain task when compared to an optimized system, how responses of components of optimized models compare to components of circuits, how parameters of the neural circuit compare to parameters of an optimized model, and how a circuit compares to an optimized model on tasks that are not directly optimized. In each case, there may be many ways to determine an optimized solution, but the optimized solution presents a single, concrete point of comparison for the neural circuit.

In this review, we begin by introducing the problem of motion estimation, including the structure placed on it by the geometry and statistics of the natural world. We then review models for motion detection that have been commonly used to describe visual circuits and computations. Finally, we consider various theoretical frameworks for studying optimization in motion detection, including how optimized models compare to measurements in diverse visual systems, including flies, the mammalian retina, and the mammalian cortex. Our goal is not to definitively answer whether motion detection systems are or are not optimized. Instead, we focus on how theories of optimality have been used to explore motion detection circuits and understand principles underlying their function.

## 2. MATHEMATICAL STRUCTURE OF VISUAL MOTION

**Figure 2:**
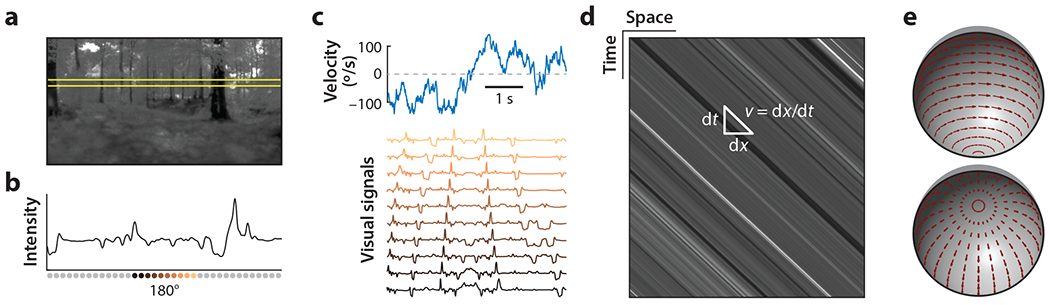
Motion detection is an inference problem. (*a*) A natural scene. (*b*) An intensity trace across the highlighted slice in panel *a*. Circles denote locations at which intensity is detected by an eye and correspond to the locations of time traces in panel *c*. (*c*) Intensity traces (*bottom*) created by an image moving with time-varying velocity (*top*). The visual system processes the intensity traces to infer the velocity. (*d*) A spatiotemporal intensity pattern created by the scene moving rightward at a constant speed. Velocity estimation is equivalent to estimating the slope of this pattern. (*e*) Self-motion creates optic flow across the retina. When an animal rotates about a vertical axis, flow is in the azimuthal direction at all elevations (*top*). When an animal translates through the world, the flow direction and speed depend on the angle with respect to the direction of movement, as well as the distance to objects (*bottom*). Panels *a–c* adapted with permission from Mano et al. (2021).

The geometry of the natural world dictates the visual signals associated with motion. Whether the visual motion is due to an animal’s self-motion or due to the motion of an external object, the relative translation and rotation of objects in the world dictate how their images pass over the retina (Figure 2). An optimized motion detection system would exploit the structure in the retinal signals to best infer the latent motion signal. In this section, we review some of the strongest structural features of retinal signals and motion signals that could be exploited through optimization.

### 2.1. Local Motion Detection

Local motion detection, or elementary motion detection, estimates the motion present in a small patch of the visual field (Hassenstein & Reichardt 1956, Yang & Clandinin 2018). It is fundamentally an inference problem, in which visual intensity signals from different locations are processed over time to estimate the local velocity, a latent variable not directly accessible in the visual contrasts (Potters & Bialek 1994, Simoncelli 1993) (Figure 2a–c). When images move on the retina, their motion can be viewed as an orientation in a space-time intensity plot (Adelson & Bergen 1985) (Figure 2d). Then, detecting local velocity is equivalent to estimating the slope of the stimulus at a particular location in space and time. As a concrete example, imagine that one has access to two of the intensity traces in Figure 2c. The goal of an elementary motion detector is to estimate the instantaneous velocity at each time from time-varying intensity measurements at these two points. An optimized detector might do this by combining the measured time traces with information about expected velocities, distributions of intensity patterns in natural scenes, and so forth.

### 2.2. Animal Translations and Rotations Result in Optic Flow

Motion across an animal’s retina is often due to its own movement. When an animal moves, the entire world rotates and translates across its eyes. These rotations and translations produce optic flow fields (Figure 2e) that jointly depend on the motion and 3D positions of the animal and other objects in the world (Koenderink & van Doorn 1987). For instance, as an animal translates forward through the world, an object directly ahead of it does not change angular position over time but does become larger, while objects to the side of the animal move across the retina. Nearby objects travel faster, sometimes occluding and then revealing more distant ones. Relative positions generally change over time.

Rotation produces the simplest form of visual motion. During rotation, all visual objects remain in the same relative position, at the same size, and there are no changes in occlusion. Instead, objects simply translocate across the retina, and the space-time orientation of local motion cues is consistent across the visual field at each angle around the axis of rotation (Figure 2d). Pure rotation is a simple case to simulate—one need simply translate an image to simulate rotation. This sort of rigid motion has been the object of many studies of optimization (see, for instance, Fitzgerald et al. 2011, Heeger & Jepson 1992, Potters & Bialek 1994).

Rao & Ruderman (1998) provided an elegant mathematical perspective on visual motion by pointing out that pure rotations form a Lie group. This means that it is possible to generate the visual stimulus at each moment in time by applying a continuous transformation to the initial stimulus. Importantly, each Lie group is uniquely specified by its generators of infinitesimal transformations, as any finite continuous transformation can be decomposed into a sequence of infinitesimal ones. The generator for pure rotations is analytically calculable, and Rao & Ruderman showed that it can be learned from data. Bahroun et al. (2019) further showed that it can be learned online from streaming data in a biologically plausible way. This generator of rotational motion can be leveraged to infer the magnitude of whole-field motion from a stream of visual inputs (Bahroun et al. 2019, Rao & Ruderman 1998). Intriguingly, Bahroun et al. showed that this first-principles computation has important similarities to both Bayes optimal motion estimators (Sinha et al. 2021) and data-driven models of the fly visual system (Salazar-Gatzimas et al. 2018).

### 2.3. From Optic Flow to Egomotion

Many animals use optic flow information to infer their own rotation and translation, or egomotion. In a classic paper, Koenderink & van Doorn (1987) studied the optimal solution for inferring self-motion from optic flow. Their analysis highlighted the importance of a wide field of view and the fundamental ambiguity between the speed of egomotion and the nearness of the visual landmarks that induce optic flow—namely, when an animal changes position and observes fast motion, it is difficult to distinguish whether the egomotion is fast or whether visual objects are just close by. An interesting feature of the optimal solution is that it must invert a nonlinear system of equations. This contrasts with the more typical linear system of equations that many use to decompose the optic flow field into templates for pure translations and rotations (see, for instance, Dahmen et al. 2001, Perrone & Stone 1994, Simoncelli et al. 1991). Various algorithms can solve these nonlinear equations (Heeger & Jepson 1992, Koenderink & van Doorn 1987).

Many visual systems have neurons selective to pure translations and pure rotations (Duffy & Wurtz 1991, Krapp & Hengstenberg 1996, Zhang et al. 2022). These feature-selective neurons could represent a biological implementation of the optic flow templates mentioned above. Some visual systems represent more varied flow fields than would be needed by a template-based approach (Zhang et al. 2022), and it is an interesting question whether this apparent redundancy allows for more accurate algorithms (Ecke et al. 2020). Biological systems also enhance their algorithms by spatially biasing optic flow estimates to those regions that have the most informative signal in the animal’s natural environment (Alexander et al. 2022, Bigge et al. 2021). They may also use different local motion detection algorithms for different types of optic flow (Creamer et al. 2018).

### 2.4. Natural Scene Statistics

Natural scenes contain a variety of statistical regularities (Simoncelli & Olshausen 2001). Motion in the scene translates these spatial regularities into spatiotemporal regularities, which can in principle serve as cues in determining the direction and speed of local motion. If a visual system has been optimized through natural selection to extract motion from natural scenes, then it would be expected to take advantage of these regularities in its optimization, and perceptual illusions could result as unexpected consequences of optimization (see the sidebar titled Shared Illusory Motion Percepts Suggest Optimized Circuits). Research into visual motion processing thereby benefits from databases of natural images and movies (Alexander et al. 2022, Salisbury & Palmer 2016, Van Hateren & van der Schaaf 1998).

**Figure 3:**
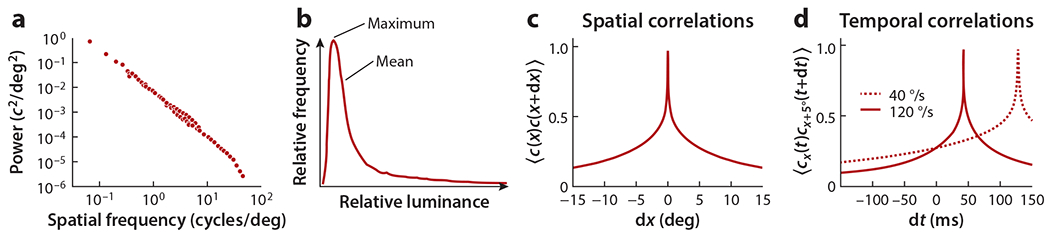
Statistical properties of natural scenes. (*a*) Power tends to be highest at low frequencies and fall off at high frequencies. Data taken from Ruderman & Bialek (1993). (*b*) Natural scenes have a positively skewed intensity distribution, so that light patches are far brighter than average, while dark patches are only a little darker than average. Data taken from Brady & Field (2000). (*c,d*) The power spectrum leads to (*c*) spatial correlations in natural scenes that become (*d*) spatiotemporal correlations when the scene moves. Panels *c* and *d* adapted from Fitzgerald & Clark (2015) (CC BY 4.0).

One important regularity in natural scenes is that neighboring samples tend to be highly correlated. If a point in a scene is bright, then neighboring points are also likely to be bright. These correlations can be represented by a correlation function over distance or, equivalently, by the power spectrum of the scene across different spatial wavelengths (Ruderman & Bialek 1993) (Figure 3a). Power spectra in natural scenes have often been fitted with power laws. These power laws have no natural length scale and imply that scenes will have similar spectra at all magnifications. When locally computed, power spectrum components can also be highly correlated across space (Schwartz & Simoncelli 2001).

A second statistical feature of natural scenes is that they are not isotropic. Orientation statistics in natural scenes have the most power along cardinal axes (up, down, left, and right), generating perceptual orientation bias in humans (Girshick et al. 2011). Along with optic flow patterns, this could account for cardinal directions of local motion detectors in the fly eye (Henning et al. 2022, Maisak et al. 2013) and direction-selective retinal ganglion cells (Sabbah et al. 2017). When artificial motion detection systems are optimized using natural scenes, this nonuniformity generates biases that align with cardinal directions and matching cortical directional distributions (Rideaux & Welchman 2020).

A third important regularity in natural scenes is their luminance asymmetry. Scenes tend to have a small number of points that are much brighter than the average across the scenes and many points that are only a little bit darker than average (Brady & Field 2000) (Figure 3b). These asymmetries can be exploited to help infer the direction and speed of motion (Chen et al. 2019, Fitzgerald et al. 2011).

When movement transforms stationary scenes into patterns over space and time, it creates a pattern in space-time that reflects the statistical regularities of the moving scene (Figure 3c,d). The regularities comprise second-order and higher-order correlations in intensity over space and time, which may be explicitly analyzed or implicitly computed using nonlinear processing steps (Fitzgerald & Clark 2015, Poggio & Reichardt 1973). An optimized motion detector should exploit the structure in these patterns of intensity.

Importantly, these image statistics are not uniform over light conditions or over spatial locations in scenes. While early visual neurons adapt to the distribution of luminances (Laughlin 1981), low-light conditions still favor integration over relatively noisier signals in early processing (Srinivasan et al. 1982) and in motion detection (Stöckl et al. 2017). Spatial differences in the statistics of visual fields influence optical flow fields, and vertebrates and invertebrates have visual circuits adapted for regional specialization in optic flow responses (Alexander et al. 2022, Bigge et al. 2021). Moreover, the statistical variability across natural scenes appears to be a limiting factor in human motion estimation (Chin & Burge 2020).

## 3. MODELS FOR LOCAL MOTION ESTIMATION

**Figure 4:**
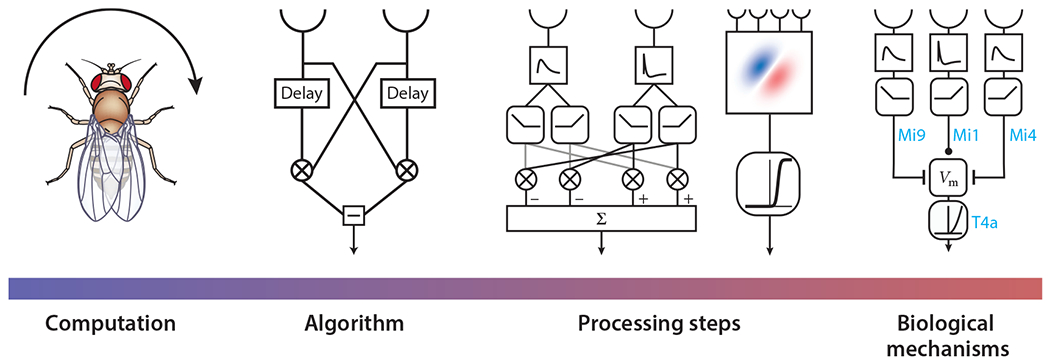
Models for motion detection occupy a continuum related to Marr & Poggio’s (1976) levels of analysis. The computational level of understanding reflects what a circuit does to promote the animal’s survival. In flies, motion detection stabilizes orientation and walking speed during navigation, among other functions. The algorithmic level reflects a mathematical summary of the computation, in this case, a correlator model, which explains fly rotational behavior very well in many, but not all, circumstances (Hassenstein & Reichardt 1956). This algorithm can be split into processing steps, which yields insight into the computation and leads to models that are progressively closer to what may be implemented in the circuit. In the figure, a linear–nonlinear model (Leong et al. 2016) and a split ON–OFF set of computations (Fitzgerald & Clark 2015, Salazar-Gatzimas et al. 2018) can be equivalent to the correlator model under some limits. Finally, the biological mechanism reflects the actual biophysical and circuit processes that implement the higher-level descriptions. In the figure, specific input neurons change conductances in a direction-selective T4 cell, a model that reduces to a correlator model with small inputs (Zavatone-Veth et al. 2020). *V*_m_ represents the T4 membrane voltage; Mi9, Mi1, and Mi4 are classes of neurons providing input to T4 at different retinotopic offsets. Images of processing steps taken from Fitzgerald & Clark (2015) (CC BY 4.0).

Models for local motion detection exist along a continuum of abstraction from explicit mechanisms in visual circuits to abstract mathematical processing steps that can be described independently of neurons or circuits. Marr & Poggio (1976) famously suggested four levels for understanding a neural computation (Figure 4): (*a*) the utility of the computation for the animal; (*b*) the algorithm describing the computation; (*c*) the constituent processing steps within that algorithm; and (*d*) the biophysical, synaptic, and other mechanisms that implement the processing in specific circuits. The third level is often neglected, but it is important because there are often many ways to implement an algorithm, and the steps arrived at by evolution are limited by the mechanisms available. We think of these identified levels as representing overlapping regions on a continuum, and real models often span across these conceptual levels. Therefore, we organize this section into subsections on algorithmic, neural network, and biophysical models for local motion detection that are not meant to follow a strict Marr hierarchy. For instance, some algorithmic models will immediately suggest processing steps and biological mechanisms, and both neural network and biophysical models can count as biological mechanisms. Nevertheless, we highlight the Marr levels in this section because they should be kept in mind throughout. Optimization of a model at one level need not imply optimization at other levels.

### 3.1. Algorithmic Models

**Figure 5:**
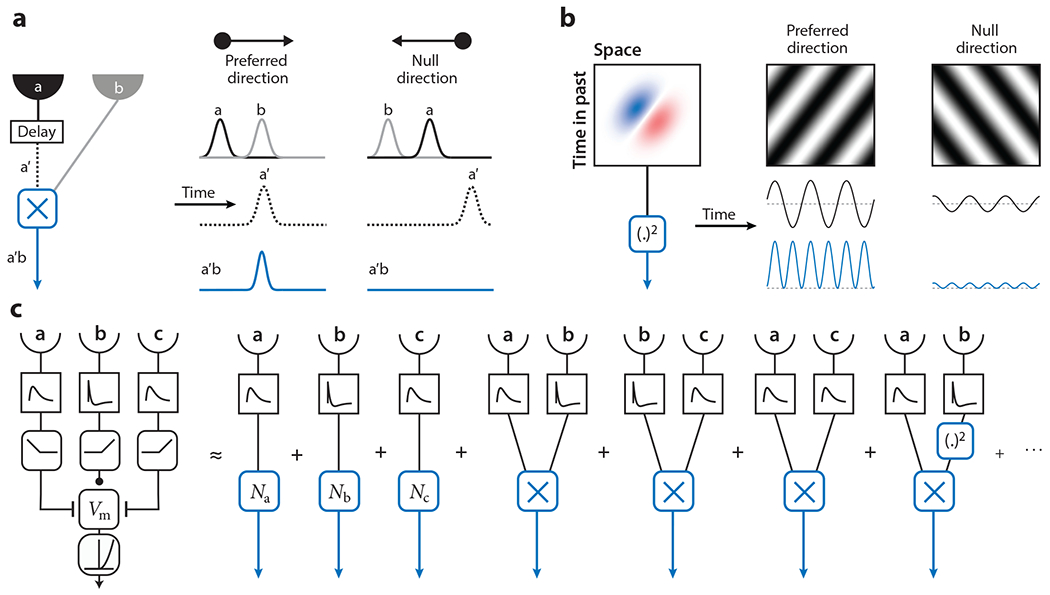
Models for motion estimation. (*a*) A correlator model of motion estimation, also known as a Hassenstein Reichardt correlator (Hassenstein & Reichardt 1956). Intensity or contrast signals from neighboring points in space are multiplied after one signal is delayed in time. This operation amplifies signals when the delayed and nondelayed signals coincide at the multiplicative step. The output of the model is the difference between two mirror-symmetric multipliers. (*b*) A motion energy model (Adelson & Bergen 1985). An oriented spatiotemporal filter amplifies signals in a preferred direction compared to the null direction, after which the filtered signal is squared. The linear operation alone does not create a direction-selective signal, since both preferred and null-direction signals have the same mean. (*c*) A biophysical model for motion estimation (Mo & Koch 2003, Zavatone-Veth et al. 2020) can be expanded into a Volterra series that approximates its operations at different polynomial orders of the input (Poggio & Reichardt 1973, Potters & Bialek 1994). The first three non-direction-selective terms each contain a nonlinearity, *N*. The lowest-order directional terms multiply pairs of inputs: These terms are approximated by correlator and motion energy models. The last term is an example third-order term, which multiplies three signals from two points in space. Other third- and higher-order terms are not shown, and we omit signs and scale factors for simplicity. *V*_m_ represents membrane voltage in the model.

At the algorithmic level, there are many frameworks to describe motion computation. These proposals represent a set of mathematical transformations that produce direction-selective signals or estimates of local velocity. In this section, we summarize the most common models and draw connections between them, since many are similar at low order (Figure 5). There need not be an obvious relationship between the algorithm and the underlying circuits (Marr & Poggio 1976); all that is required is that the algorithm approximate the circuit output.

#### 3.1.1. Gradient model.

When images are rigidly translated, they generate intensity derivatives in both space and time. The gradient model states that, in such a case, the image’s velocity can be written as *ν* = *∂_t_C/∂_x_C*, where *C* is the local intensity, and *∂_x_* and *∂_t_* are the partial derivatives with respect to space and time (Fennema & Thompson 1979). The gradient model serves as the basis for models that fit partial derivatives to estimate optic flow fields (see, for instance, Simoncelli et al. 1991). The gradient model is also the solution to an optimal model for motion detection in the limit of high signal to noise (Potters & Bialek 1994). Nonetheless, this model has several problems in its implementation. First, it takes perfect partial derivatives in space and time, when, in fact, spatial measurements are irreducibly discrete due to discrete photoreceptors, and temporal derivatives cannot be computed instantaneously. The gradient model’s estimate degrades when measurements of partial derivatives contain noise and the denominator approaches 0. These derivative estimates can be optimized to account for noise and the discreteness of spatial sampling (Farid & Simoncelli 2004).

#### 3.1.2. Correlator model.

The correlator model was first proposed to explain directional visual behavior in insects (Hassenstein & Reichardt 1956) (see Figure 5a), but it also explains a wide variety of primate psychophysical data (Van Santen & Sperling 1985). It multiplies pairs of signals to compute pairwise correlations in intensity or contrast between adjacent points in space that are differentially filtered in time. Subtracting a mirror-symmetric signal yields a signed correlation that provides information about the direction and speed of local motion, especially once averaged over time or space. In this model, the spatial filters at the inputs and the temporal filters that precede multiplication can be optimized and will depend strongly on the statistics of motion (Fitzgerald et al. 2011, Potters & Bialek 1994).

The correlator model does not produce an estimate of the true velocity, since its performance depends on the structure of the moving scene. For instance, its mean output depends on the temporal frequency of a drifting sinusoidal grating (Egelhaaf & Borst 1989), not its velocity, a counterintuitive prediction that matches neural responses and behavior in several animals (Creamer et al. 2018, Haag et al. 2004, Yildizoglu et al. 2020). However, correlator models may better approximate the stimulus velocity for broadband natural images (Dror et al. 2001, Fitzgerald & Clark 2015), and early visual processing and correlator mechanisms in the fly brain may be jointly adapted to the statistics of natural images (Chen et al. 2019, Dror et al. 2001).

Motion detection requires a nonlinear interaction over time and space (see the sidebar titled Motion Detection Requires a Nonlinear Operation). The correlator model approximates the lowest-order nonlinear interaction term (Poggio & Reichardt 1973), acting as a low-order approximation of a wide range of motion detection models. This helps explain its broad applicability to motion detection. It is also the optimal model at low signal-to-noise ratios (SNRs) (Potters & Bialek 1994).

#### 3.1.3. Motion energy model.

In the motion energy model, input images are convolved with oriented space-time filters, after which outputs are squared, and mirror-symmetric signals are subtracted (Adelson & Bergen 1985) (Figure 5b). With specific choices of spatiotemporal filters, this model is identical to the correlator model, and any motion energy model may be written as the sum of correlator models. Both the motion energy model and the correlator model estimate motion by relying exclusively on pairwise correlations in intensity. The energy framework has the convenient property that the model’s average response to a stimulus is the weighted sum of the stimulus’ spatiotemporal power spectrum, which provides powerful intuition about the model.

The motion energy model has become prevalent in explaining both neural and psychophysical data because the intermediate computational steps taken in the model appear to match neural processing steps. In particular, in the cortex, in the retina, and in the fly visual system, there are directional cells with strong, oriented, linear receptive fields similar to those in this model (De Valois et al. 2000, Fransen & Borghuis 2017, Leong et al. 2016, McLean & Palmer 1989, Rust et al. 2005, Salazar-Gatzimas et al. 2016). This correspondence between algorithm and mechanisms means that any optimization of the model’s spatiotemporal linear filters would have direct cellular correlates.

#### 3.1.4. Higher-order correlations.

A motion estimator, *r*, can be thought of as a functional, *F*, acting on a visual stimulus, **s**, such that the estimator is *r* = *F*[**s**]. This functional can be expanded into a series of Volterra kernels (Figure 5c):

$$r\left(t\right)=K^{\left(0\right)}+\underset{i}{\sum }\int \text{d}\tau K_{i}^{\left(1\right)}\left(\tau \right)s_{i}\left(t-\tau \right)+\underset{i,j}{\sum }\int \text{d}\tau_{1}\int \text{d}\tau_{2}K_{i,j}^{\left(2\right)}\left(\tau_{1}-\tau_{2}\right)s_{i}\left(t-\tau_{1}\right)s_{j}\left(t-\tau_{2}\right)+\underset{i,j,k}{\sum }\int \text{d}\tau_{1}\int \text{d}\tau_{2}\int \text{d}\tau_{3}K_{i,j,k}^{\left(3\right)}\left(\tau_{1},\tau_{2},\tau_{3}\right)s_{i}\left(t-\tau_{1}\right)s_{j}\left(t-\tau_{2}\right)s_{k}\left(t-\tau_{3}\right)+\cdots ,$$

where the stimulus, *s_i_*(*t*), depends on both space and time. In this expansion, *K*^(1)^ acts as a linear kernel, *K*^(2)^ acts as a quadratic kernel, etc. In this framework, the constant and linear terms are both non-direction-selective (see the sidebar titled Motion Detection Requires a Nonlinear Operation), so that the quadratic term is the lowest-order term that can define motion direction. The Volterra series formulation relates naturally to higher-order spike-triggered methods for systems identification but is distinct (Bialek & de Ruyter van Steveninck 2005, Schwartz et al. 2006). The Bayes optimal motion estimator can be written as a Volterra series that computes a variety of higher-order correlations that depend on the statistics of the visual world, its motion, and the noise level (Fitzgerald et al. 2011, Potters & Bialek 1994).

The correlator and motion energy models discussed above are both low-rank approximations of the *K*^(2)^ term, relying exclusively on pairwise correlations to estimate motion direction and speed. However, second-order correlations are just the lowest-order correlation that contains information about speed and direction. Higher-order correlations also provide useful cues that can be used to estimate motion (Fitzgerald et al. 2011). Triplet correlations—those among three points in space and time—exist in natural scenes (Clark et al. 2014, Nitzany & Victor 2014) and are processed as motion signals by humans, monkeys, fish, and flies (Clark et al. 2014, Hu & Victor 2010, Nitzany et al. 2017, Yildizoglu et al. 2020). These triplet correlations interact with pairwise correlations in local motion estimates (Chen et al. 2019, Nitzany et al. 2016). Correlator and motion energy–like models can access terms beyond the second order via simple extensions that modify the form of the nonlinearity (Fitzgerald & Clark 2015, Taub et al. 1997). The form of the kernels, *K*^(*i*)^, at different orders is strongly constrained by the structure of the motion detector. In this framework, a suite of Volterra kernels {*K*^(*i*)^} could be optimized, including by using low-rank approximations of the kernels.

### 3.2. Neural Network Models

Neural network models for motion detection tend to focus on the processing steps for motion detection, one level down from the algorithms discussed above, but some also reference low-level biological mechanisms. They use linear filtering and point nonlinearities, along with architectures that mirror those in visual motion circuits.

#### 3.2.1. Linear–nonlinear models.

One of the most commonly applied neural models is the linear–nonlinear (LN) model, in which a signal is linearly filtered before it is acted on by a point nonlinearity. In the context of motion detection, these models use an oriented filter in space-time, as does the motion energy model, which amplifies the amplitude of signals in the preferred direction compared to the null direction. Note that it is only after the nonlinearity is applied that the mean signals in the preferred direction are larger than in the null direction (see the sidebar titled Motion Detection Requires a Nonlinear Operation). Nonlinearities in these classes of models can be chosen for their simplicity or fitted to data (Heeger 1991, Leong et al. 2016, Rust et al. 2005). In principle, the chosen nonlinearity can make models sensitive to all manner of spatiotemporal correlations beyond the second-order ones in the motion energy model (Fitzgerald & Clark 2015, Hu & Victor 2010). LN models have been suggested to underlie direction selectivity in flies (Leong et al. 2016, Wienecke et al. 2018), the mammalian retina (Kim et al. 2014), and the mammalian cortex (Jagadeesh et al. 1993). The linear filtering in such models is supported by various data (Jagadeesh et al. 1993, Wienecke et al. 2018), and the shape of the filter can be measured in directional cells (De Valois et al. 2000, Fransen & Borghuis 2017, Leong et al. 2016, McLean & Palmer 1989, Rust et al. 2005, Salazar-Gatzimas et al. 2016). LN models are often grouped with the motion energy model, which they somewhat resemble. In LN models, however, one could optimize both the linear filter and the form of the point nonlinearity, whereas many properties that are important in the motion energy model follow from its quadratic nonlinearity.

The Barlow-Levick model was first proposed as a logic-based mechanism to explain directional signals measured in the rabbit retina (Barlow & Levick 1965). In a continuous formulation, it can be written as a rectifying nonlinearity acting on summed excitation and delayed inhibition from neighboring points in space, making it a widely applied class of the LN model. In this model, motion in the null direction activates the delayed inhibition to veto the subsequent excitatory signals; in the preferred direction, the excitation beats the inhibition, and a signal is passed on. When expanded to low order, this model is equivalent to a single multiplier in the correlator model (Clark & Demb 2016).

#### 3.2.2. Cascade models.

In circuits that detect motion, early motion signals are combined and weighted by downstream neurons in a cascading processing scheme. In models of cortical motion detection, this cascading processing of motion signals is critical. In these models, motion signals first appear in cortical area V1, at which point they are tuned to the local components of visual motion. In area MT, these local motion signals are combined to generate signals that are selective for the direction of pattern movement (Rust et al. 2006, Simoncelli & Heeger 1998). Similarly, in the mammalian retina, direction-selective retinal ganglion cells pool directional signals from a population of starburst amacrine cells, so that models of ganglion cell responses are cascade models (Schachter et al. 2010, Wei 2018). In insects, wide-field neurons downstream of local motion detectors similarly pool visual signals, often to increase selectivity to specific types of egomotion (Krapp & Hengstenberg 1996, Mauss et al. 2015). Likewise, models for cortical neurons selective for egomotion flow fields pool directional inputs across visual space (Perrone & Stone 1994).

#### 3.2.3. Recurrent networks.

Biological circuitry is not feedforward, and it is interesting to ask how recurrent network mechanisms could contribute to visual motion processing. One possibility is that motion selectivity can arise in recurrent networks that function to predict sequential data (Pachitariu & Sahani 2012, Rao & Sejnowski 1999). The neural coding of visual motion in recurrent networks might therefore provide insight into the normative concepts of predictive coding and predictive information (Palmer et al. 2015, Srinivasan et al. 1982). Recurrent networks can also emerge as biologically plausible implementations of algorithms for visual motion processing (Bahroun et al. 2019).

#### 3.2.4. Normalization.

Models of motion detection tend to suffer from a dependence on stimulus strength. For instance, in a correlation model, doubling the stimulus contrast yields a response that is four times larger, even when the stimulus is moving at the same velocity. This problem is also likely experienced by biological motion detectors, which tend to respond more strongly to higher-contrast stimuli. One solution, for models and biological circuits alike, is to normalize signals. This can be accomplished upstream of the motion detector, where visual circuits adjust their gain to account for changing input contrasts (Baccus & Meister 2002, Rieke 2001). Such normalization has been shown computationally to improve the fidelity of motion detection (Drews et al. 2020, Matulis et al. 2020). Normalization can also occur downstream of motion detectors, where divisive normalization of signals has been proposed to account for a host of measurements in cortical motion signals (Carandini & Heeger 2012, Simoncelli & Heeger 1998).

### 3.3. Biophysical Models

Biophysical models have proposed a variety of low-level mechanisms that could underlie motion detection. For instance, these models can map the delays, summations, and nonlinearities in the algorithmic and neural network models discussed above onto synapses, conductances, calcium channels, and spiking mechanisms in direction-selective circuits. Conductance-based models for motion detection can create multiplication-like operations and oriented linear receptive fields (Torre & Poggio 1978). In flies, conductance models have been proposed to explain the responses of directional cells to a wide variety of different stimuli (Badwan et al. 2019; Gruntman et al. 2018, 2019; Zavatone-Veth et al. 2020), to explain changes in tuning with altered delay dynamics (Gonzalez-Suarez et al. 2022), and to provide mechanisms for multiplication-like phenomena in these cells (Borst 2018, Groschner et al. 2022). In the retina, biophysical models have been proposed to explain independent signals in the neurites of starburst amacrine cells (Poleg-Polsky et al. 2018), as well as the origins of their direction selectivity based on internal processing (Hausselt et al. 2007) or on relative delays among inputs and a postvoltage nonlinearity (Ding et al. 2016, Srivastava et al. 2022). In the cortex, conductance-based neuron and network models have been used to account for response properties in direction-selective cortical neurons (Mo & Koch 2003, Suarez et al. 1995). Biophysical models tend to have a large number of parameters that could, in principle, be optimized.

## 4. INSIGHTS FROM THEORIES OF OPTIMIZATION

Most of the models described above are descriptive models of motion detection circuitry. These descriptive models can represent the workings of visual circuits at various levels of abstraction from the low-level details. However, to understand not just how the circuit works, but also why evolution has settled on these specific solutions, another view of these models is necessary. Instead of asking how they fit to the data, one may ask how a model would be parameterized if it were optimized in some way, for instance, to best detect direction or image velocity. These then become normative models—what models should look like if they are optimized for specific tasks.

### 4.1. Prelude on Encoding, Decoding, Objective Functions, and Constraints

**Figure 6:**
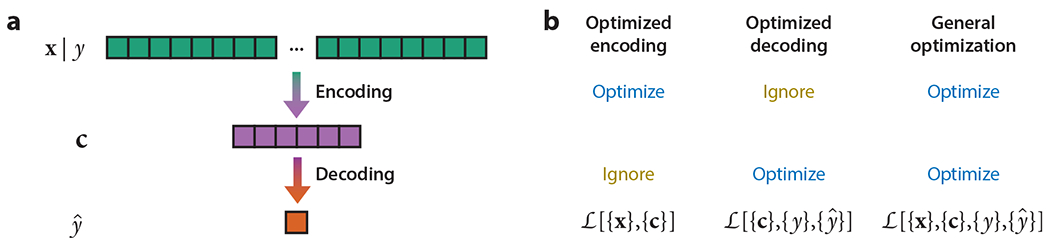
The many meanings of optimal. (*a*) The visual system computes intermediate representations, **c**, of the input **x**: **x** ↦ **c** ↦ *ŷ*. Although only one is shown, there could be several layers of representations. Encoding is moving from **x** to **c**, while decoding is moving from **c** to a useful quantity *ŷ*, which is latent in **x** and **c**. (*b*) Some theories restrict optimization to the encoding or decoding step, constraining or ignoring the other step. Other theories impose fewer constraints and/or use a loss function *ℒ* that depends on both input and output representations. Many different optimization theories can be derived by choosing different objective functions and constraints.

Neural representations both encode their inputs and also set limits on downstream decoding (Figure 6a). This makes it easy to debate whether representations are optimized for encoding or for decoding. From the encoding point of view, the system’s goal is to maintain information about its input. Its accuracy is often quantified with Shannon information. From the decoding point of view, the goal of the system is not just to maintain information, but rather to output a direct quantitative estimate of a feature of interest. Its accuracy is often quantified with the mean squared error.

Encoding and decoding frameworks can make different predictions about what quantities will be encoded within the biological system. For instance, both the speed and direction of motion must be encoded to perfectly specify the velocity of left–right motion. If leftward and rightward motion are equally likely, then it takes one bit to specify the direction of motion, but many more bits may be required to specify the scalar value of speed. From the viewpoint of encoding information, it is more beneficial to accurately encode the speed of motion than its direction. On the other hand, a system that only encodes motion speed is terrible for decoding the velocity because the expected root mean squared error would be a factor of root two larger than the true speed. This performance is worse than a system that always reports the velocity to be zero. From the viewpoint of decoding velocity, the one bit associated with the motion’s direction is more valuable than the many bits associated with speed.

However, it would be a mistake to dwell too long on the false dichotomy between optimizing for encoding and optimizing for decoding. From an abstract point of view, both are simply optimization problems with different objective functions and constraints (Figure 6b). It is likely that neither the Shannon information nor the mean squared error accurately models the quantity that biological systems fundamentally care about, and the implied constraints are too naive. It is interesting and important to figure out what optimization objectives and constraints best explain the phenomenology observed in the biological system. We consider several possibilities below. A theory of optimality derived for these empirically determined objective functions and constraints would likely provide the most insight into the biological system, transcending both the encoding and decoding frameworks.

### 4.2. Optimization Frameworks for Modeling Visual Motion Processing

It is typically impossible to find error-free computations in noisy nonlinear systems. However, fundamental principles of information processing can be used to discern how the system could best approximate the desired computation. These principles from information processing are far more general than any specific biological system, but remarkably, these principles often reveal optimal solutions that provide quantitative insights into real biological systems. It is particularly impressive that the brain can achieve near-optimal computations despite architectural constraints and the noise inherent to biological wetware. Building models that explicitly incorporate these constraints can help researchers to better understand the algorithms and mechanisms that underlie brain computation.

In this section, we review several optimization frameworks that have been useful in explaining how biology performs visual motion estimation. The subsections are loosely ordered to start with basic stimulus encoding and increasingly add computational goals related to behavior and structural constraints related to neural circuit architecture.

#### 4.2.1. Efficient coding.

Efficient coding is one of the oldest and most commonly applied optimization frameworks to study visual computation with normative modeling (Attneave 1954, Barlow 1961, Simoncelli & Olshausen 2001). In its simplest forms, efficient coding quantifies information without regard to the information’s quality or behavioral utility, making it well suited for understanding generalist coding strategies in early visual circuits (Atick & Redlich 1990, Bell & Sejnowski 1997, Van Hateren & van der Schaaf 1998). Motion-sensitive neurons can arise in generally efficient representations when motion is a prominent component of the stimulus being encoded (Li 1996). Moreover, efficient coding predicts motion encoding that is interestingly correlated with other visual features, such as the size of the receptive field and color selectivity, providing a normative perspective on the conjunctive coding of neurons in the primary visual cortex (Li 1996).

A core tenet of efficient coding theory is that neurons should adapt their coding strategies to the statistics of sensory stimuli (Atick & Redlich 1990, Barlow 1961, Laughlin 1981). Motion is lower dimensional than many other visual signals, and this has made motion-encoding neurons useful model systems for studying the principles of neuronal adaptation (Brenner et al. 2000, Fairhall et al. 2001, Reisenman et al. 2003, de Ruyter van Steveninck et al. 1994). Understanding how these adaptive phenomena result from fixed nonlinearities and/or dynamic processing is an interesting area of theoretical research (Borst et al. 2005, Safran et al. 2007).

#### 4.2.2. Bayes optimal estimation.

von Helmholtz [2013 (1924)] argued that visual systems combine sensory evidence with experience to generate percepts, or best guesses about the world. This view fits closely with a Bayesian view of motion estimation, in which sensory evidence is the likelihood, and the experience is represented by a prior (possibly generated over evolutionary time). If Bayesian models fit behavioral experiments, then that in itself is evidence for optimization, since the Bayesian method for combining the prior with the likelihood is optimal. Both the likelihood and prior functions can also be optimized for performance; we consider these cases below.

A Bayesian view of velocity estimation has proven adept at explaining human velocity percepts, especially as the SNR of the sensory data is experimentally altered. According to this theory, lower-contrast images provide lower-signal-to-noise evidence about visual motion, so that the prior exerts more influence on the final percept. This can explain percepts of both direction and speed in humans (Stocker & Simoncelli 2006, Weiss et al. 2002). These effects rely on a prior for low-speed motion across the retina, which seems sensible since most objects in the world are stationary. Although the prior and likelihood functions are not themselves optimized, this theory argues that the percepts are themselves optimal, since they optimally combine sensory data with prior knowledge of velocity distributions. These sorts of models are often also called ideal observer models.

One very interesting model for motion estimation considered Gaussian velocity, contrast, and noise statistics to explicitly derive the Bayes optimal estimator of stimulus velocity (Potters & Bialek 1994). The optimal estimator depended strongly on the SNR of the inputs to the system. In the limit of high SNR, the optimal estimator resembled a gradient model, while in the limit of low SNR, the optimal estimator resembled a correlator model. These two models make different predictions for the tuning to different velocities and wavelengths of sinusoidal drifting gratings. These predictions were directly tested in the fly by measuring neurons responding to drifting sinusoidal gratings at a range of contrasts and luminances, representing both low and high SNRs in the system (Haag et al. 2004). That experimental test found that the tuning remained correlator-like even as the SNR of the stimulus was increased substantially, suggesting that, in the fly, there is not a switch in estimator behavior with increasing SNR. One explanation for this result is that it may be difficult to evolve an estimator that can switch forms with changes in input noise. Alternatively, under natural conditions, the shot noise associated with luminance and contrast, tested in this experiment, may be dominated by noise associated with sampling natural scenes with different contrasts and spatial correlations. If this alternative source of noise dominates, then it may be that high-SNR environments do not exist naturally. Although this optimal model does not appear to predict the behavior of the fly’s motion detectors, it remains informative because it demonstrates that the fly has evolved a system distinct from the model’s view of optimality, which requires exploration and explanation.

Many models of motion estimation assume a stimulus prior that is symmetric with respect to contrast, a simplifying assumption but one that is at odds with natural scene statistics (see Section 2.4). One Bayes optimal model for motion detection did not make this assumption and predicted that an optimal motion estimator should incorporate third-order correlations into its motion estimates (Fitzgerald et al. 2011). Indeed, humans, flies, and fish perceive motion in binary stimuli containing third-order correlations (Clark et al. 2014, Hu & Victor 2010, Yildizoglu et al. 2020). Moreover, a simple extension to a correlator model can permit responses to higher-order stimulus correlations. When such a model is trained to estimate motion in natural scenes, it responds to third-order stimuli with tuning similar to that in flies and fish (Fitzgerald & Clark 2015, Yildizoglu et al. 2020), although humans respond in a different pattern (Clark et al. 2014, Hu & Victor 2010). A different, related experiment extracted second- and third-order kernels for fly behavior from uncorrelated stimuli, as in the equation in Section 3.1.4 (Chen et al. 2019). Simulations showed how second- and third-order cues could be combined to improve estimates of natural scene motion. In the experiments discussed above, the theory of optimal motion computation drove experimenters to use asymmetric stimuli, and visual responses were interpretable in light of the theory.

#### 4.2.3. Information bottleneck.

Computational goals and constraints can sometimes be incorporated into a model in a theoretically principled manner. Vision operates with limited bit rates (Koch et al. 2006), and it is generally impossible to find an encoding that contains only relevant information. The information bottleneck theory quantifies the maximal amount of information that a computational system can encode about a variable of interest, given constraints on the total amount of information that it retains about its inputs (Tishby et al. 2000). The information bottleneck theory thereby quantifies the minimal information overhead and provides a framework for understanding both task-relevant and task-irrelevant information coding. It reveals diminishing returns in information processing, so that computational systems may sometimes encode less information than is possible because encoding more comes at the cost of representing an excessive amount of irrelevant information.

The information bottleneck theory has been successfully applied to visual processing, especially predictive information coding (Palmer et al. 2015, Sachdeva et al. 2021). In this work, the important concept is that the visual system should selectively encode information that is relevant for future stimuli, but it may need to encode seemingly irrelevant information about the past to achieve this goal. The retina is impressively close to the information-theoretic limit (Palmer et al. 2015). The motion of visual stimuli has clear relevance to predicting future visual stimuli, and quantifying predictive information with the information bottleneck method has also helped researchers to understand noncanonical ways that the circuitry in the primate retina encodes motion (Liu et al. 2021, Manookin et al. 2018). The information bottleneck has also been used to demonstrate the benefits of gap junctions for self-motion coding and predictive information coding in the fly visual system (Wang et al. 2017, 2021).

#### 4.2.4. Motion estimation in closed-loop behavior.

Motion vision and motion-guided behavior are tightly tied together in sensorimotor feedback loops. Visual motion estimation allows animals to detect optic flow that often indicates their own motion through the environment (Section 2.2). As a consequence, many animals respond to optic flow with compensatory actions that reduce the optic flow, with optomotor (Hassenstein & Reichardt 1956, Naumann et al. 2016) and optokinetic (Kretschmer et al. 2017, Portugues et al. 2014) responses controlling the body and eyes, respectively. Indicative of this tight sensorimotor coupling, many motion-sensitive neurons in the visual system are modulated by the behavioral state of the animal (Chiappe et al. 2010, Saleem et al. 2013).

Our understanding of the logic of visual motion coding could benefit from optimization models that incorporate the closed-loop impact of behavior. For instance, control theory provides insight into how the coding of visual motion relates to behavioral demands and motor control (Holman et al. 2023, Markov et al. 2021, Yang et al. 2022). Several information-theoretic frameworks have also been proposed to quantify how sensory encoding should be adapted to match the demands of ongoing behavior (Buckley et al. 2017, Still 2009, Tishby & Polani 2010), but these have not yet been applied to visual motion processing.

#### 4.2.5. Performance optimization.

With a parameterized model, one may ask whether optimizing it to perform some task also predicts parameters or other metrics that match the biological system (Figure 1). These models may not be globally optimal, but their task-optimized model parameters have proven useful for understanding many visual circuits (Turner et al. 2019, Yamins & DiCarlo 2016).

For the mouse retina, convolutional neural network models constrained by the connectivity of the retina have been trained to predict the direction of motion of random dot patterns (Murray et al. 2022). Interestingly, when trained in this task, nodes in the network emerge with functional properties and interactions that resemble cell types in the direction-selective retinal circuit (starburst amacrine cells and direction-selective retinal ganglion cells).

For the cortex, other models constrained by visual system architecture have been optimized to detect motion direction and speed. One such model fitted linear filters in an artificial neural network to best estimate the direction of naturalistic flow (Rideaux & Welchman 2020). The study found that the optimization yielded populations of nodes with cardinal direction biases matching those in cortical neurons, as well as nodes that integrate local motion signals similar to neurons in cortical area MT. A different study instead optimized the linear filtering in a visual system–like artificial neural network to best estimate both the direction and speed of motion (Burge & Geisler 2015). This optimized network had similar performance to humans in several psychophysical tasks, including speed discrimination.

For flies, parameterized neural network models of motion detection have been optimized to accurately predict the motion of natural scenes. In one case, correlation-type detectors were modified to allow them to detect third- and fourth-order correlations (Fitzgerald & Clark 2015). When the model was trained to predict velocities of rigidly translating natural scenes, it predicted fly-like behavioral responses to triplet correlations (Clark et al. 2014). In a second case, model motion detectors trained to predict scene velocity recapitulated asymmetric responses to light and dark stimuli (Leonhardt et al. 2016). A third study using a correlator-type motion detector and parameterized signal normalization showed how normalization could improve motion estimation over the variability among different natural scenes (Drews et al. 2020).

In flies, comprehensive connectomic data provide strong constraints on models of motion detection. One study incorporated virtually all available connectomic data to constrain a model of the fly’s early visual processing (Lappalainen et al. 2023). It trained networks of scores of neuron types with connectomically constrained connections to predict optic flow in natural movies. Importantly, the constraints and training led to models that correctly identified direction-selective local motion detectors within the eye network, correctly identified ON and OFF channels in the eye, and suggested new targets for study based on novel predicted properties. A different study incorporated a far smaller set of neurons that serve as input to directional neurons (Mano et al. 2021). The model was optimized to accurately predict velocity or direction. Perhaps surprisingly, the optimized model possessed many functional features of the real motion circuit, which were not predicted by first principles: It split into ON and OFF motion channels, possessed the measured polarity and relative delays of inputs to the motion detectors, exhibited responses to stationary features, and generated decorrelation between channels. The model best matched the biology when there was high noise during optimization, suggesting that noise in the circuits may be critical for understanding the organization of this motion circuit.

## 5. CONCLUSIONS

Modern approaches to neuroscience provide stunning experimental possibilities and a dizzying amount of data. They can generate dense reconstructions of neural circuits and measure functional responses from tens of thousands of neurons. Measurements of calcium, voltage, and synaptic release can be made in a variety of animal states in response to any visual stimulus. With these capabilities, there are many response properties that can be measured in the visual system, but not all of them will be important for specific questions of interest, and it is notoriously challenging to construct a systems-level understanding by studying the parts alone (Anderson 1972, Churchland & Sejnowski 1988, Marr & Poggio 1976). For instance, is early lateral inhibition among photoreceptors important for velocity estimation or not?

Optimization, and its focus on system performance, can help to answer this sort of question (Biswas et al. 2020, Richards et al. 2019, Sterling & Laughlin 2015). If optimizing the system to perform a task results in a biological feature appearing in the optimized model, it provides a compelling argument that that feature is contributing to the system performance. This provides a path for using optimization to make sense of large-scale data, and optimality principles can even be applied as priors that regularize statistical analyses of data (Młynarski et al. 2021). In the most extreme case, one could ask what set of target functions, constraints, and learning rules result in all of the features measured in the circuit, when optimized. This is clearly easier said than done. Evolutionary and biological constraints are difficult to ascertain, as are the true targets of evolution. For instance, has a direction-selective cell evolved to best estimate direction, velocity, or some nonlinear function of velocity? What metric defines “best”? This sort of approach must involve trying out different targets and constraints to see when optimization results in biology-like models. Optimization with constraints tells us about sufficiency conditions to predict features of circuits, but it remains difficult to know how wide this set of sufficient conditions is. It is thus unclear how far one can push this approach, but optimization need not have universal applicability to be a powerful principle (Biswas et al. 2020).

For these reasons, the right question is not, Is this circuit optimal? This sort of question has an artificially binary answer, and too much is hidden in the definition of optimal. For example, the answer is almost certainly no for any optimization function that we can currently write down and almost certainly yes for a coerced notion of optimality that includes everything that matters biophysically, developmentally, and evolutionarily. More interesting questions are the following: How does this circuit relate to an optimized one? What does optimization explain? In answering this second class of questions, there is a rich set of constraints to explore and many ways to think about optimality. The comparison between the biology and each optimized model can provide insight into the meaningful features of biological circuits and suggest constraints on circuit function.

## MOTION DETECTION REQUIRES A NONLINEAR OPERATION

By definition, direction-selective signals have different mean responses to stimuli moving in different directions. This cannot result from purely linear operations on the stimulus (Adelson & Bergen 1985, Borst & Egelhaaf 1989). To illustrate this, consider a 1D linear model written as a convolution in time and space,

$$r\left(x,t\right)=\int \text{d}\theta \int \text{d}\tau K^{\left(1\right)}\left(\theta ,\tau \right)S\left(x-\theta ,t-\tau \right),$$

where *r*(*x, t*) is the model response at each point in space and time, *K*^(1)^ is the linear kernel, and *S*(*x, t*) is the stimulus. The mean response (over time and space) is

$$\langle r\rangle =\langle S\rangle \int \text{d}\theta \int \text{d}\tau K^{\left(1\right)}\left(\theta ,\tau \right).$$

Since this mean response depends on the stimulus only through its mean, left- and right-moving versions of the same stimulus produce identical mean responses.

Beyond examining the mean of the response, one could ask if a linear model’s responses to stimuli moving in opposite directions have different maxima or different amplitudes. Such metrics, while easy to perform visually, implicitly use nonlinear operations to judge direction selectivity.

## SHARED ILLUSORY MOTION PERCEPTS SUGGEST OPTIMIZED CIRCUITS

Illusions occur when our percepts are different from our reasoned perceptual expectation. For instance, stationary patterns of contrasts or colors can elicit strong motion percepts (Fraser & Wilcox 1979, Kitaoka & Ashida 2003). Rather than being algorithmic failures, illusions can often be interpreted as incorrect inferences in systems that are optimized for statistics that are distinct from the illusory stimulus. This approach has been used to explain direction and speed percepts that change with object contrast (Stocker & Simoncelli 2006, Weiss et al. 2002).

When evolutionarily divergent animals share an illusory motion percept, it suggests that their motion circuits may be similarly optimized. For instance, many animals, from flies to fish to primates, perceive motion in the same kinds of stationary luminance patterns (Agrochao et al. 2020, Gori et al. 2014, Kitaoka & Ashida 2003). Moreover, the reverse phi illusion is common to beetles, flies, fish, and primates (Anstis 1970, Hassenstein & Reichardt 1956, Orger et al. 2000) (see **Supplemental Video 1**), and motion percepts in response to higher-order correlations can be similar or distinct among animals (Clark et al. 2014, Hu & Victor 2010, Yildizoglu et al. 2020). In all of these cases, algorithms optimized for natural scene performance predict the illusions as a consequence of computations that are useful in natural settings (Agrochao et al. 2020, Fitzgerald & Clark 2015, Watanabe et al. 2018). These algorithmic similarities in evolutionarily distant animals suggest strong selective pressure toward those specific algorithms—namely, optimization.

The circuit mechanisms and neurons underlying illusory percepts can increasingly be uncovered experimentally (Agrochao et al. 2020; Conway et al. 2005; Livingstone & Conway 2003; Salazar-Gatzimas et al. 2016, 2018), illuminating how the response properties of individual neurons relate to optimized processing.

## SUMMARY POINTS

1. Natural selection suggests that visual systems and motion detection are optimized to perform well. Optimized models provide a useful point of comparison for the properties and performance of neural circuits.
2. Motion estimation provides a rich substrate for optimization because it is requisitely nonlinear; it can exploit the geometrical structure of motion, as well as regularities of natural scenes; and it involves multiple computational stages, from estimating local motion signals to inferring egomotion from optic flow.
3. Models for motion estimation range from first-principles to data-driven ones, and from algorithmic to biophysical ones. They span many animal models and parts of the brain. Each can, in principle, be compared to optimized versions.
4. Many different optimized models for motion detection have been compared to circuits and behavior, showing both similarities and differences with visual system measurements. For both similarities and differences, one learns about the sufficiency of different loss functions and constraints to reproduce visual system properties.

## FUTURE ISSUES

1. Theories of optimization have tended to focus predominantly on either stimulus encoding or decoding. Future work could profitably move toward jointly optimizing neural activity for both encoding and decoding.
2. Connectomic data offer a powerful constraint on the architecture of neural network models, and it will be important to figure out how best to incorporate such data into models.
3. It will be important to develop connections between optimizing at the performance level and at the circuit or biophysical level.
4. Common constraints considered during optimization have involved noise levels, available nonlinearities, and network architecture. Going forward, it is important to explore the impact of energy constraints and realistic biophysics.
5. Future optimization protocols could close the behavioral feedback loop, so that the loss function incorporates the performance of the motion detector embedded in naturalistic animal behaviors.
